# Understanding the mechanism of binding between Gab2 and the C terminal SH3 domain from Grb2

**DOI:** 10.18632/oncotarget.19323

**Published:** 2017-07-18

**Authors:** Angelo Toto, Daniela Bonetti, Alfonso De Simone, Stefano Gianni

**Affiliations:** ^1^ Istituto Pasteur - Fondazione Cenci Bolognetti, Dipartimento di Scienze Biochimiche “A. Rossi Fanelli” and Istituto di Biologia e Patologia Molecolari del CNR, Sapienza Università di Roma, 00185, Rome, Italy; ^2^ Department of Life Sciences, Imperial College London, SW7 2AZ, London, UK

**Keywords:** binding kinetics, folding, induced fit, intrinsically disordered proteins, mutagenesis

## Abstract

Gab2 is a large disordered protein that regulates several cellular signalling pathways and is overexpressed in different forms of cancer. Because of its disordered nature, a detailed characterization of the mechanisms of recognition between Gab2 and its physiological partners is particularly difficult. Here we provide a detailed kinetic characterization of the binding reaction between Gab2 and the C-terminal SH3 domain of the growth factor receptor-bound protein 2 (Grb2). We demonstrate that Gab2 folds upon binding following an induced fit type mechanism, whereby recognition is characterized by the formation of an intermediate, in which Gab2 is primarily disordered. In this scenario, folding of Gab2 into the bound conformation occurs only after binding. However, an alanine scanning of the proline residues of Gab2 suggests that the intermediate contains some degree of native-like structure, which might play a role for the recognition event to take place. The results, which represent a fundamental step forward in the understanding of this functional protein-protein interaction, are discussed on the light of previous structural works on these proteins.

## INTRODUCTION

The Grb2-associated binding protein 2 (Gab2) is 74 kDa scaffolding protein that is involved in functional cellular signalling and cancer development [1–4]. It is composed of large disordered regions and various structural domains and docking sites that act as a platform for the assembly of several protein-protein interactions [5]. While lacking any enzymatic activity, Gab2 is a direct mediator of receptor tyrosine kinases and non- receptor tyrosine kinases, such as cytokine and G-protein–coupled receptors, transmitting and amplifying signals to downstream effectors [6–8]. In fact, upon stimulation, Gab2 activates and binds to several targets involved in signal transduction and has a role in a variety of cellular functions, such as proliferation, survival, migration, and differentiation [9–11].

Gab2 is implicated in a number of cancers of both solid and haematological origin. In particular, Gab2 is overexpressed in breast [12], gastric [13] and lung [14] cancers. Furthermore, its role is clearly established in haematological cancers, like juvenile myelomonocytic leukaemia [15], chronic myelogenous leukemia (CML) [16], acute leukaemia [17] and acute lymphoblastic leukaemia [18]. Importantly, whilst the functions of Gab2 are critical in the embryonic and postnatal development, its expression in a healthy mature cell is relatively suppressed. For these reasons, targeting Gab2 provides a promising route to develop new strategies for highly selective drug therapies in anticancer treatment. In fact, this protein acts at the head of signalling networks, by conveying inputs from membrane receptors to the cytoplasm, where it dictates the functions of multiple pathways.

From a structural perspective, the N-terminal part of Gab2 consists of a Pleckstrin Homology domain (of about 120 amino acids), which binds the lipid phosphatidylinositol-3, 4, 5-triphosphate of the plasma membrane and is critical for the membrane localization of the protein [5], whereas the remainder of the protein (whose total length is 676 amino acids) essentially lacks any defined secondary or tertiary structure, such that Gab2 may be classified as an intrinsically disordered protein (IDP).

The disordered regions of Gab2 have a fundamental functional relevance, by promoting the direct interaction with a number of cytoplasmic partners containing SH2 or SH3 domains [10, 19–21]. Typically, upon binding to their specific partners, the disordered regions of Gab2 encounter a disorder-to-order transition, as exemplified for example in [22]. Because of this complexity, being the IDPs typically elusive to a mechanistic characterizations [23], the detailed mechanisms of interactions between Gab2 and its physiological partners have remained elusive.

**Figure 1:**
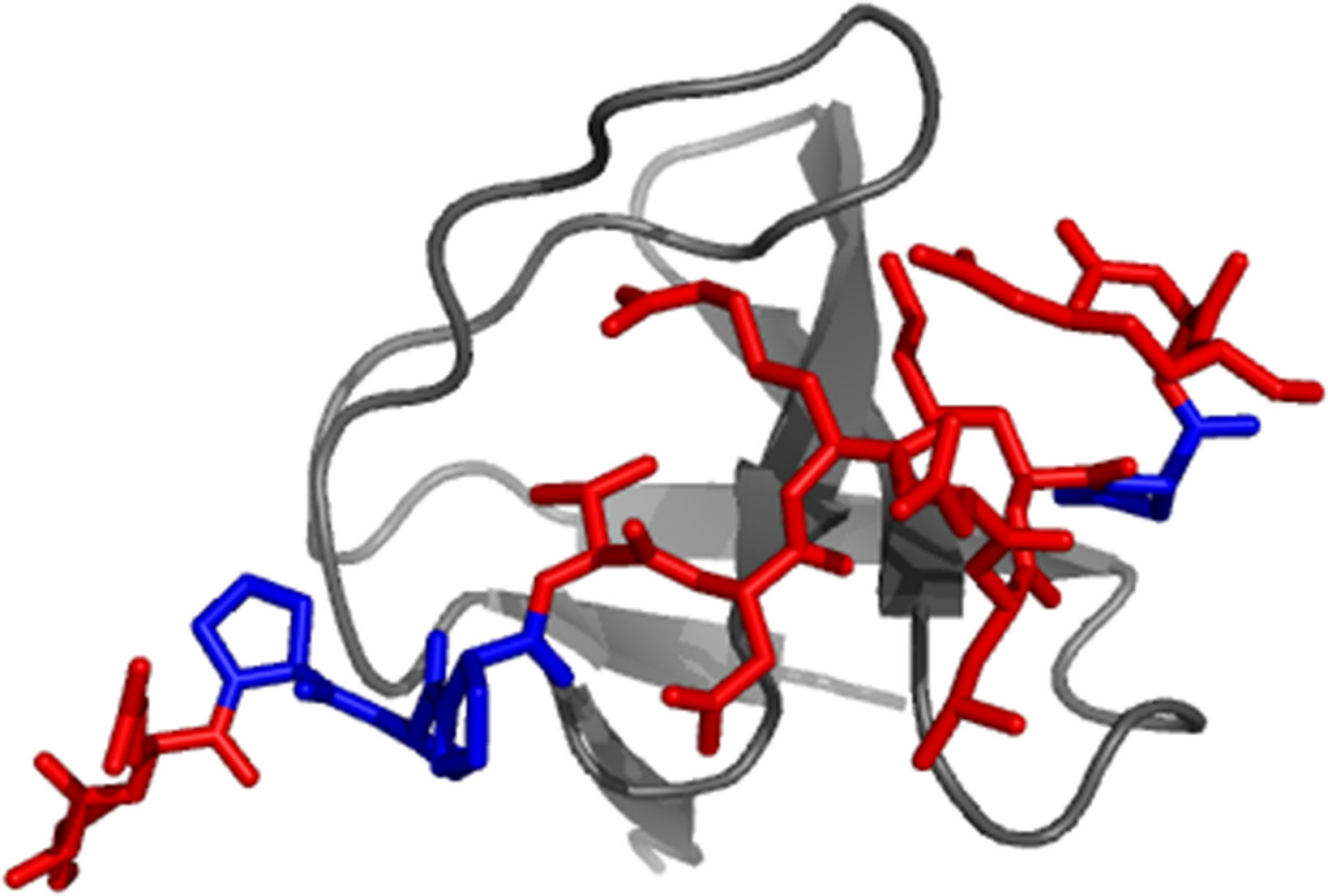
Cartoon representation of the Gab2-Grb2 SH3C complex Three-dimensional structure of the complex between the C-terminal SH3 domain of the Grb2 protein (Grb2 SH3C) and a peptide representing the residues 503-524 of the Grb2-associated binding protein 2 (Gab2_503-524_). The SH3 domain is represented in gray while the peptide is represented in red. The four proline residues of Gab2_503-524_ are highlighted in blue. The picture was generated using the software PyMOL (pdb code: 2vwf). Crystallization conditions were 50mM HEPES pH 7.5, 50mM NaCl (peptide:protein 3:1 molar ratio); reservoir: 0.7 M tri-sodium citrate, 0.1 M Tris pH 8.5.

One of the critical physiological interactions involving Gab2 lies in the binding with Grb2 [19]. This recognition occurs between the C-terminal SH3 domain of the latter protein (Grb2 SH3C) and a region of Gab2 encompassing residues 503-524 (Gab2_503-524_) [24], which is predominantly disordered in isolation whereas it adopts a polyproline type II fold upon binding (Figure 1) [24]. Interestingly, in its unbound state Gab2_503-524_ has a degree of propensity to adopt conformations resembling the Grb2-bound state [22]. Whilst the structural features of Gab2_503-524_ has been described both in its free state and when bound the Grb2 SH3C [24], little is known about the mechanism of recognition between these two elements. Characterising this mechanism of recognition is of great importance for understanding both the molecular basis underlying the role of Gab2 in functional and pathological processes and for the general structural basis of protein-protein interactions by IDPs. In this work, we address this important open question by providing a detailed kinetic characterization on the recognition between Gab2_503-524_ and Grb2 SH3C. Binding kinetics, measured both in the μs and ms time range, were obtained by monitoring intrinsic fluorescence using both a stopped flow and temperature jump techniques. In agreement with what expected from a disordered system [25, 26], data reveal the presence of complex kinetics, which enabled the identification of at least two kinetic steps. We show that a comparison between the experiments performed by varying the concentration of Gab2_503-524_ or Grb2 SH3C reveals unambiguously that the reaction occurs via an induced fit type mechanism, whereby the folding of Gab2_503-524_ to a polyproline type II structure is subsequent to the formation of an initial encounter complex, despite its partial character of polyproline II when in the unbound state. Furthermore, by performing an alanine scanning of the four proline residues of Gab2_503-524_, we observe that the association rate constant between the two molecules correlate with the stability of the polyproline type II structure, an observation that supports the view that the initial encounter complex, whilst not fully folded, contains some native-like secondary interactions, as also suggested by the NMR analysis of the isolated fragment [22].

## RESULTS AND DISCUSSION

Understanding the mechanism of binding between two molecules requires first to establish the simplest kinetic scheme describing the reaction adequately and then to infer the mechanistic details [27]. Accordingly, the rational of our experimental efforts to unveil the recognition between Gab2_503-524_ and Grb2 SH3C was focused first on unveiling the order of events and subsequently on understanding the main features of the reaction.

### A folding after binding mechanism describes the recognition between Gab2_503-524_ and Grb2 SH3C

An intrinsically disordered protein, such as Gab2_503-524_, may explore a disorder-to-order transition upon binding to its physiological partner. In these cases, from a mechanistic perspective, it may be argued that the reaction could occur *via* two extreme mechanisms, namely conformational selection, formally equivalent to a Monod−Wyman−Changeaux model, [28] or folding after binding, equivalent to an induced fit model [29]. Although conformational selection requires the IDP to populate at least partly the folded state in the free form, i.e. as observed for Gab2_503-524_ [22], the pre-existence of ordered structures in IDPs does not necessarily commit to a conformational selection mechanism, and kinetic studies are needed to address these questions [30].

**Figure 2:**
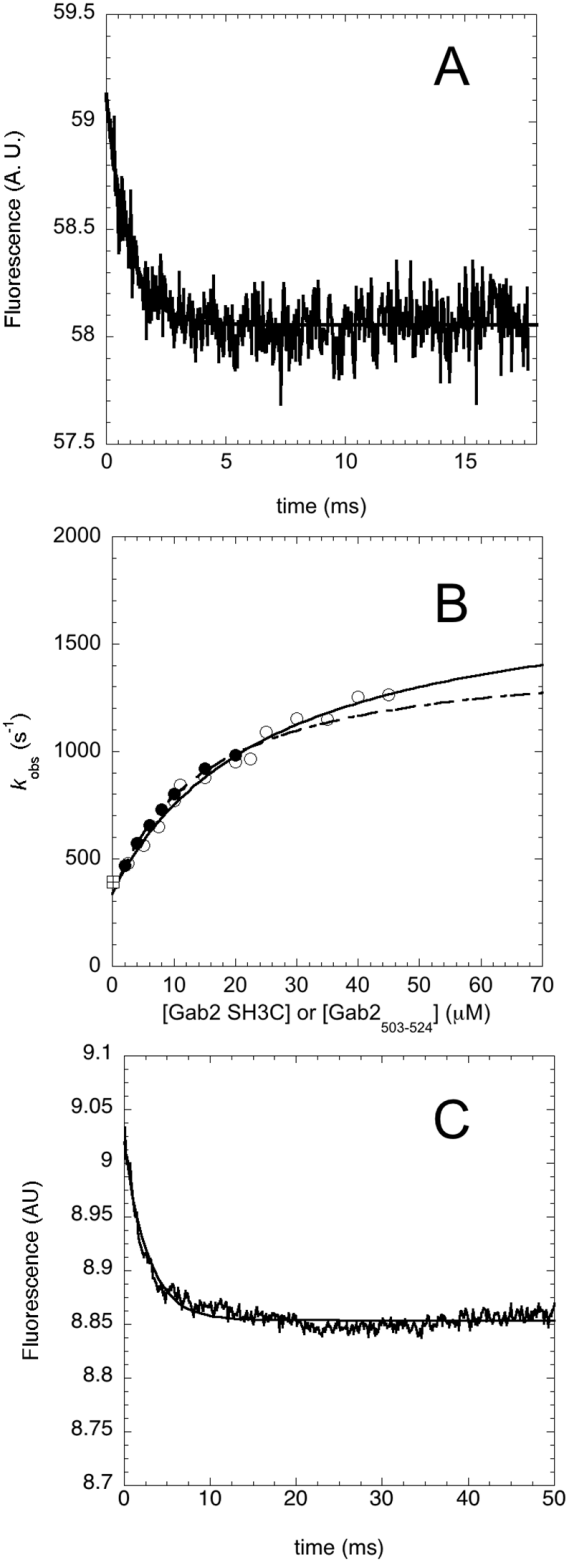
Binding kinetics between Grb2 SH3C and Gab2_503-524_ performed by using a temperature jump apparatus at 25°C **(A)** Typical temperature jump time course obtained in a (un)binding experiment between Grb2 SH3C 4 μM and Gab2_503-524_ 15 μM. Line is the best fit to a single exponential decay. **(B)** Plot of the observed relaxation rate constants as a function of reactant concentration. Pseudo-first-order binding experiments were performed by keeping the concentration of Grb2 SH3C at 4 μM and varying the concentration of Gab2_503-524_ (empty circles), and keeping the concentration of Gab2_503-524_ at 2 μM and varying the concentration of Grb2 SH3C (filled circles). In both cases the observed rate constants follow a hyperbolic behavior. The square symbol refer to the rate constant obtained from displacement experiments. **(C)** Displacement experiment. A pre-incubated complex of wild type Grb2 SH3C (10 μM) and Gab2_503-524_ (10 μM) was rapidly mixed with an excess of Grb2 SH3C variant W35Y/W36Y. The line is the best fit to an exponential decay.

In an effort to understand the order of the events in the folding reaction of Gab2_503-524_ upon binding to Grb2 SH3C, we resorted to apply a kinetic test, originally introduced by Olson et al [31] and more recently applied on different protein systems [32–34]. This experimental strategy is essentially based on the comparison of the observed binding kinetics when performing pseudo-first order experiments with respect to each ligand. Thus, we performed relaxation binding experiments between Gab2_503-524_ and Grb2 SH3C by temperature jump. In particular, a constant concentration of Grb2 SH3C was mixed with different excess concentrations of Gab2_503-524_ and the resulting solution was subjected to a rapid increase of temperature of 9°C, from 16 to 25°C, using capacitor-discharge temperature-jump instrument. Similarly, we performed binding experiments by keeping the concentration of Gab2_503-524_ constant and varying the concentration of an excess of Grb2 SH3C. Binding was observed by monitoring the change in fluorescence of Grb2 SH3C, which is primarily ascribed to residues W35 and W36. A typical temperature jump trace is reported in Figure 2A. Under all the experimental conditions here considered, the binding time courses were consistent with a single exponential decay. While this observation, to a first approximation, may be consistent with a two-state mechanism, a detailed analysis of the observed rate constants, as shown below, revealed an additional level of complexity.

A plot of the observed relaxation rate constants as a function of reactant concentration is reported in Figure 2B. The experiments performed under pseudo-first order conditions of the two different reactants provide data that are in both cases consistent with a hyperbolic dependence. This finding suggests that the observed kinetics is more complex than a simple two-state model, where a linear behavior would be expected, and at least a bimolecular and monomolecular step may be detected. For the nature of the reaction, we postulate these steps to be ascribed to a mechanism of binding and subsequent folding of Gab2_503-524_ into a polyproline type II structure. Since both experiments provide very similar values of observed rate constants, it may be concluded that the reaction occurs via an induced-fit type mechanism, whereby Gab2_503-524_ tends to recognize Grb2 SH3C in a relatively unstructured state and folding follows binding. In fact, it has been previously demonstrated that, due to its symmetry with respect to the reactants, only the induced fit scenario can account for a hyperbolic behavior when performing pseudo-first order experiments with respect to both ligands [31, 32, 34]. In contrast, in the case of conformational selection mechanism, an approximately linear dependence of the observed rate constant would have been observed when the concentration of Gab2_503-524_ was higher than the concentration of Grb2 SH3C.

In order to validate further the kinetic parameters obtained from pseudo-first-order experiments, we resorted to measure the dissociation rate constants with an additional method. In analogy to classical experiments on heme proteins [35], we carried out displacement experiments where a pre-incubated complex between two partners was challenged with an excess of a competing reactant. Thus, we performed displacement by producing a double mutant variant of Grb2 SH3C where the two fluorescent tryptophan residues were replaced with two tyrosines (W35Y/W36Y). Because W35Y/W36Y has a very different fluorescence yield compared to wild type Grb2 SH3C, a displacement between the pre-formed complex triggered by a rapid mix with an excess of W35Y/W36Y could be readily monitored. The observed dissociation rate constant was found insensitive to concentration of all the reactants and provided a value of *k*_*off*_ = 350 ± 30 s^-1^. As shown in Figure 2, this value is consistent with what extrapolated from relaxation experiments.

### Addressing the mechanism of recognition between Gab2_503-524_ and Grb2 SH3C by mutagenesis

**Figure 3:**
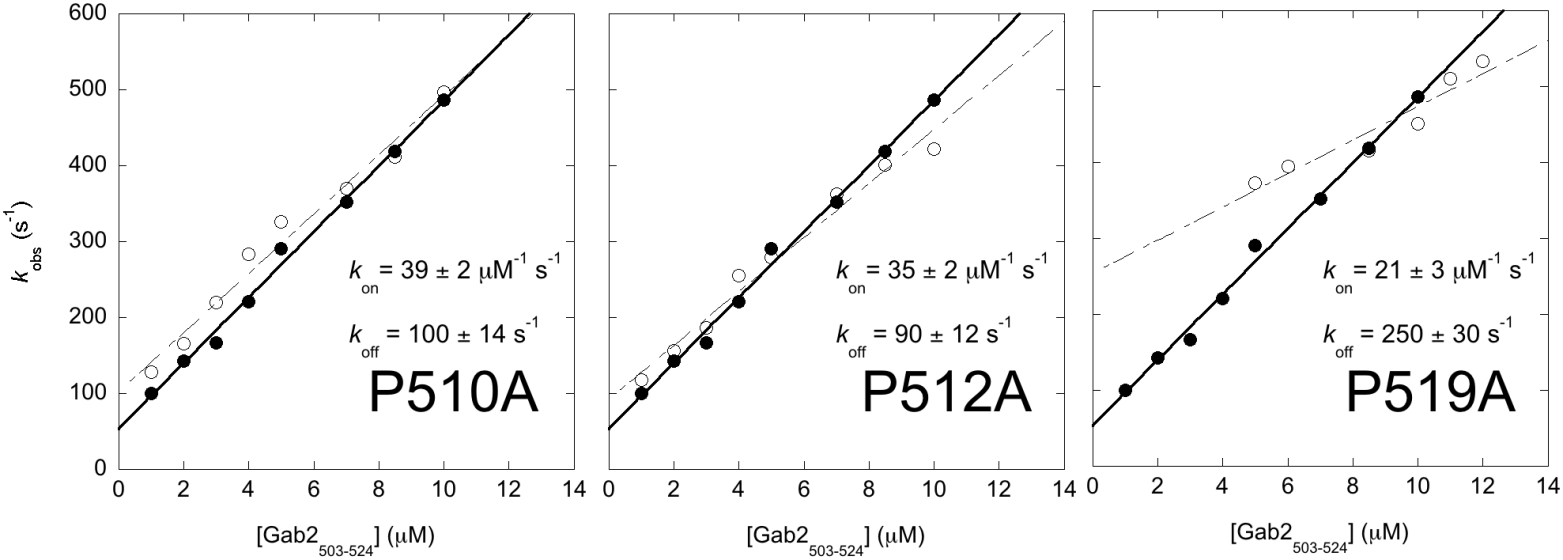
Alanine scanning of the proline residues of Gab2_503-524_ measured by using a stopped-flow apparatus at 10°C The observed rate constants are plotted as a function of increasing Gab2_503-524_ concentration, while keeping Grb2 SH3C at a constant concentration of 0.5 μM. In each graph both Gab2_503-524_ wild-type (filled circles) and mutant (empty circles) are shown. Mutant P511A was not shown because it was not possible to measure any detectable binding. The observed rate constants follow a linear dependence from which the association (*k*_*on*_) and dissociation (*k*_*off*_) rate constants can be calculated. The calculated *k*_*on*_ and *k*_*off*_ for Gab2_503-524_ wild-type are 43 ± 1 μM^-1^ s^-1^ and 53 ± 7 s^-1^ respectively.

It has been recently suggested that the proline residues of Gab2_503-524_ play a critical role both in tuning the preformed structure of the isolated protein and in modulating the affinity for Grb2 SH3C [22]. Thus, in an effort to understand directly the role of this position in the reaction mechanism, we carried out an alanine scanning of the four proline residues of Gab2_503-524_ and characterized the binding kinetics of the variants P510A, P511A, P512A and P519A. With the exception of P519, none of the other proline residues appear to participate directly to binding by establishing direct contacts with Grb2 SH3C. In order to compare the effect of mutagenesis more accurately, we performed these experiments by reducing the experimental temperature to 10°C and, therefore, measuring reaction kinetics using a stopped-flow apparatus. In these conditions, whilst we could not explore the full hyperbolic dependence of the observed rate constants (being too fast at high reactant concentrations for the stopped-flow apparatus), we could measure the apparent association and dissociation rate constant with higher precision. Importantly, control experiments with wild type Gab2_503-524_ show that the rate constants obtained with stopped-flow methodology were consistent with those obtained by temperature jump. Whilst for the variant P511A we could not measure any detectable binding, the observed pseudo-first-order rate constant for the P510A, P512A and P519A variant are reported in Figure 3. From the measured parameters a K_D_ of 2.5 ± 0.2 μM, 2.5 ± 0.1 μM and 12 ± 1 μM could be calculated for P510A, P512A and P519A respectively.

It is of interest to comment on the magnitude of the association rate constants of the different variants (Figure 3). In fact, earlier NMR analysis of the same variants suggested a clear ranking of WT > P510A > P512A > P519A > P511A in the tendency of populating preformed Grb2-bound like structures [22]. Accordingly, investigation of the data in Figure 3 suggests a similar trend when the association rate constant is taken into account. In particular, the lower the tendency of Gab2_503-524_ to explore pre-formed structure, the lower its rate constant of association to Grb2 SH3C. This finding appears to contrast our observation that the recognition between the two molecules occurs via a pure induced fit mechanism and suggests that some embryonic native-like secondary structure might be present in the initial encounter complex. On the basis of these observations, we suggest that, whilst the overall kinetics of binding is consistent with an induced fit scenario, at a molecular level a mixed mechanism may occur. More specifically, we propose that some pre-formed bound-like conformations are adopted by the disordered Gab2_503-524_, which confers some features of conformational selection. Thus, some native-like structure might be present already at the early stages of binding with Grb2 SH3C.

## CONCLUSIONS

A comprehensive understanding of the binding between Gab2_503-524_ and Grb2 SH3C, a crucial interaction in normal cellular signalling for a number of cancer conditions, demands the full description of their mechanism of recognition. By performing a combined stopped-flow and temperature jump kinetic study, we successfully demonstrated that these two molecules bind through a initial encounter complex, whose characterization is potentially very important to the identification of molecules able to hijack this interaction and therefore to inhibit the aberrant functions of Gab2 in a number of solid and haematological cancers. Whilst the mechanism is formally similar to an induced fit scenario, the analysis of variants tuning the residual structure in Gab2_503-524_ indicates that the initial complex contains some embryonic bound-like secondary structure that funnels the binding energy landscape thereby effectively accelerating the formation of the macromolecular complex. Future work based on extensive site directed mutagenesis will shed light on the detailed structural features of such a metastable complex.

## MATERIALS AND METHODS

### Protein expression and purification

The sequence encoding for the Grb2 SH3C domain, subcloned in a pET28-b+ plasmid was used to transform E.coli cells (BL21). Expression of the protein was obtained growing bacterial cells in LB medium, at 37°C until they reached OD_600_ = 0.6-0.8 and then induced with IPTG at the concentration of 1mM at 37°C over night. Cells were then collected through centrifugation.

To purify the protein, pellet was resuspended in buffer TrisHCl 50mM, NaCl 0.5M, pH 7.5, with the addition of antiprotease tablet (Roche), then bacterial cells were lysed by sonication and centrifuged. The lysate was loaded onto a Nickel-charged Hi-trap Chelating column (GE Healthcare) equilibrated with buffer TrisHCl 50mM, NaCl 0.5M, 10mM Imidazole, pH 7.5 and eluted with a gradient from 10mM to 1M Imidazole using an ÄKTA-prime system. Fractions containing the purified protein were analysed through SDS-page and collected. Buffer was then exchanged with 25mM HEPES, 100mM potassium acetate, pH 7.5.

The Gab2_503-524_ peptide in its wild-type form (SRGSEIQPPPVNRNLKPDRKAK) and its variants were purchased from JPT, Germany. Peptide concentrations were measured by weighting 1 mg of the sample in an analytical balance and concentration was further confirmed by measuring absorbance at 205 nm.

### Temperature-jump binding experiments

Kinetic experiments were performed using a PTJ-64 capacitor-discharge T-jump apparatus (TgK Scientific). Temperature was rapidly changed with a jump-size of 9°C, from 16°C to 25°C. The buffer used was 25mM HEPES, 100mM potassium acetate, pH 7.5, the excitation wavelength was 296 nm, and the fluorescence emission was collected using a 320-nm cut-off glass filter. Binding experiments were performed by keeping Grb2 SH3C concentration constant at 4 μM and varying the concentration of Gab2_503-524_ from 2.5 μM to 45 μM, and keeping Gab2_503-524_ concentration constant at 2 μM and varying the concentration of Grb2 SH3C from 2 μM to 20 μM. All the observed relaxation rate constants obtained and analysed were calculated from the average of 10 individual traces.

### Stopped-flow binding experiments

Binding kinetics experiments were carried out on a single-mixing SX-18 stopped-flow instrument (Applied Photophysics); the excitation wavelength used was 280 nm and the fluorescence emission was collected using a 320-nm cut-off glass filter. Pseudo-first order binding experiments were performed mixing a constant concentration of Grb2 SH3C (0.5μM) versus increasing concentration of Gab2_503-524_, from 1μM to 10μM, in its wild-type form and its variants P510A, P511A, P512A, and from 1μM to 12μM for the P519A variant.

For all binding experiments the temperature was 10°C and the buffer used was 25mM HEPES, 100mM potassium acetate, pH 7.5. The observed rate constants were calculated from the average of 3-6 single traces. Association rate constants (*k*_*on*_) were calculated as the slope of a linear function fitting the observed rate constants versus ligand concentration, while dissociation rate constants (*k*_*off*_) were calculated either from extrapolation as the intercept of y-axis, or from separate displacement experiments.

### Stopped-flow displacement experiments

The dissociation rate constant was measured by carrying out displacement experiments on a single-mixing SX-18 stopped-flow instrument (Applied Photophysics); the excitation wavelength used was 280 nm and the fluorescence emission was collected using a 320-nm cut-off glass filter. A pre-incubated complex of wild type Grb2 SH3C (10 μM) and Gab2_503-524_ (10 μM) was rapidly mixed with an excess of Grb2 SH3C variant W35Y/W36Y (100 μM and 200 μM). Displacement experiments were performed at 25°C, in presence of 25mM HEPES, 100mM potassium acetate, pH 7.5. The observed rate constants were calculated from the average of 5 single traces. Observed kinetics was consistent with a single exponential decay.
